# Impact of oral health on the quality of life of public servants: an analysis of associated factors

**DOI:** 10.47626/1679-4435-2024-925

**Published:** 2024-09-24

**Authors:** Vitória Freitas de Araújo, Ismael Lima Silva, Layla Beatriz Barroso de Alencar, Samara Crislâny Araújo de Sousa, Ozanna Soares Medeiros de Araújo, Cristiano Moura

**Affiliations:** Center for Rural Health and Technology, Universidade Federal de Campina Grande, Patos, PB, Brazil

**Keywords:** health evaluation, oral health, quality of life, occupational groups, universities, avaliação em saúde, saúde bucal, qualidade de vida, categorias de trabalhadores, universidades

## Abstract

**Introduction:**

Oral health encompasses functional and aesthetic aspects that directly impact an individual’s daily activities.

**Objectives:**

To analyze the impact of oral health conditions and related factors on the quality of life of public servants.

**Methods:**

This cross-sectional study was conducted with a sample of 89 public servants, both permanent and outsourced, from a federal public university in northeastern Brazil. Outcome was the impact of oral health on daily activities, determined by the Oral Impacts on Daily Performance indicator, and independent variables were sociodemographic characteristics, normative oral health conditions, access to and use of dental services, self-reported oral morbidity, self-perception of oral health, and absenteeism because of dental reasons. All data were analyzed using descriptive and analytical statistics, using the chi-squared test for heterogeneity and linear trend. The significance level used was set at 5%, with a 95% CI.

**Results:**

Participants experienced at least one day of work impaired by dental problems in the six months prior to the survey. Prevalence of dental absenteeism was 7.9%. Variables associated with the impact of oral health on daily activities included gender, toothache, toothache intensity, self-reported oral health, awake bruxism, self-reported need for dentures, number of natural teeth in the upper and lower arches, and signs of temporomandibular dysfunction.

**Conclusions:**

Sociodemographic, clinical, and subjective oral health variables influence the impact of oral health on the daily activities of public servants.

## Abstract (pt)

**Introdução:**

A saúde bucal consiste em aspectos funcionais e estéticos que impactam diretamente nas atividades diárias dos indivíduos.

**Objetivos:**

Analisar o impacto das condições de saúde oral e seus fatores associados na qualidade de vida de servidores públicos.

**Métodos:**

Este estudo foi realizado de forma transversal, com amostra composta por 89 servidores, efetivos e terceirizados, de uma universidade pública federal da região Nordeste do Brasil. O desfecho foi o impacto da saúde bucal nas atividades diárias, determinado pelo indicador Oral Impacts on Daily Performance, e as variáveis independentes foram características sociodemográficas, condições normativas de saúde bucal, acesso e utilização de serviços odontológicos, morbidade bucal referida, autopercepção da saúde bucal e absenteísmo por motivos odontológicos. Todos os dados foram analisados pela estatística descritiva e analítica, usando o teste de qui-quadrado de heterogeneidade e tendência linear. O nível de significância utilizado foi de 5%, e o intervalo de confiança, de 95%.

**Resultados:**

Os trabalhadores tiveram, pelo menos, um desempenho diário afetado por problemas odontológicos nos 6 meses anteriores à pesquisa. A prevalência de absenteísmo por motivos odontológicos foi de 7,9%. As variáveis que estiveram associadas ao impacto da saúde bucal nas atividades diárias foram o sexo, a dor de dente, a intensidade da dor de dente, a autoavaliação da saúde bucal, o bruxismo de vigília, a autoavaliação da necessidade de prótese, o número de dentes naturais na arcada superior e inferior, assim como indícios de disfunção temporomandibular.

**Conclusões:**

Variáveis sociodemográficas, clínicas e subjetivas em saúde bucal influenciam o impacto da saúde bucal nas atividades diárias de servidores públicos.

## INTRODUCTION

Oral health should be understood as a comfortable, functional, aesthetically pleasing dentition that enables individuals to perform not only their daily activities but also their social function without physical and psychological impairment.^1^ In this context, work is a fundamental and highly relevant factor for social integration, such that working conditions affect individuals’ quality of oral health, potentially causing mucosal changes, trauma, and other oral cavity problems.^2^ Therefore, individuals or population groups in need of oral health promotion, prevention, and rehabilitation^1^ need to be identified.

Subjective indicators can be used to measure the impact of oral health on workers’ quality of life.^2^ This approach allows for the assessment of oral health, public policy planning, access to health services, and treatment plans to be based not only on the biological parameters of the disease.^1,3,4^

Of the subjective indicators used, the Oral Impacts on Daily Performances (OIDP)^5^ is considered one of the most comprehensive because it quantifies the impact of oral health conditions on an individual’s daily activities in terms of frequency and severity. In addition, this indicator allows for the measurement of an individual’s perception of this impact, as well as an assessment of its causes, and can be used in the planning and evaluation of health programs.^6^

However, besides measuring the impact of oral health on daily activities and reflecting on quality of life, understanding that there are multidimensional issues at play is critical.^1,7^ Thus, individual demographic factors (e.g., age and color/race), predisposing and facilitating factors (e.g., education, income, self-reported general and oral health), oral health status factors, and self-perceived need for treatment factors comprise a model of multiple explanatory predictors of the impact of oral health on an individual’s daily activities and quality of life.^1,4,6,8^

The aim of the present study was to assess the oral health conditions, and the factors associated with the impact of these conditions on the daily activities of public servants at Centro de Saúde e Tecnologia Rural, Universidade Federal de Campina Grande (CSTR/UFCG).

## METHODS

The present study was characterized as observational, analytical, individual and cross-sectional. The convenience sample consisted of 89 public servants, both permanent and outsourced, at CSTR/UFCG in the city of Patos, state of Paraíba, Brazil. All participants present on the campus during the survey period were included, while those who refused to participate or did not sign the Informed Consent Form (ICF) were excluded from the sample.

Researchers were trained to act as examiners through theoretical training by using technical manuals and video lessons, and practical training by using photographs of clinical conditions. These photographs simulated possible clinical conditions that the investigators might face during data collection. In addition, this training phase aimed to ensure consistent interpretation, understanding, and application of the questionnaire and physical examination. Internal consistency and inter- and intraexaminer reliability were assessed using kappa coefficient (k) and inter-and intraexaminer percentage agreement for the following clinical conditions: tooth loss as well as use and need for dentures. The results showed 97% inter-and intraexaminer agreement and k = 0.94.

Prior to the actual data collection, a pilot study was conducted with the following objectives: to evaluate the quality of the data collection instrument, especially regarding the comprehensibility of the questionnaire; to evaluate the fieldwork methods (interview duration and minimum number of interviews per shift); and to perform clinical calibration with the necessary adjustments for the data collection phase. For this study, 20 randomly selected public servants at CSTR/UFCG were administered the questionnaire and underwent a physical examination.

An interview and physical examination, conducted by an interviewer trained for this task, were conducted at the worker’s site either in the morning or in the afternoon. The physical examinations were conducted in open areas under natural light. Disposable wooden spatulas, a ballpoint periodontal probe (Community Periodontal Index/World Health Organization [CPI/WHO] probe), and a properly sterilized dental mirror were used. The examiner performed the physical examination with all necessary personal protective equipment (PPE), such as a cap, mask, gloves, goggles, and/or face shield.

Quality control was performed by repeating the interview and physical examination in a random sample of 10% of the participants. Ak = 0.92 was found for the clinical conditions evaluated in this control.

The dependent variable used was “impact of oral health on daily activities.” This variable was assessed using the OIDP instrument, validated for Brazilian adults and older individuals.^9^ This instrument comprises nine questions related to daily performance, namely: eating, speaking, brushing teeth, exercising, mood, social relationships, feeling embarrassed, studying and working, and sleeping. Each question was preceded by the question, “Some people have problems that may be caused by their teeth. Which of the following situations apply to you in the past six months?” Response options were yes/no.

Sociodemographic variables were gender, age, marital status, race/color, and education. In terms of oral health prevention, the following variables were considered: seeking dental care (in the past 12 months), location of the last dental visit, reason for the last dental visit, evaluation of the last dental visit, attendance at the UFCG School of Dentistry Clinic, self-efficacy score for nightly toothbrushing, and dental-related absenteeism.

In addition, the following subjective and clinical oral health conditions were assessed: toothache (past 6 months); intensity of toothache; pain in the face, sides of the head, cheeks, or in front of the ears (past 6 months) and intensity of this pain; self-reported oral health; self-reported need for dental treatment; awake bruxism; use of dentures; self-reported need for dentures; number of natural teeth in the upper and lower arches; and symptoms of temporomandibular dysfunction (TMD) by using the Fonseca anamnestic index.^10^

Descriptive statistics using absolute and relative frequencies were used to characterize the sample. For inferential statistics, bivariate analysis was performed using the chi-square test for heterogeneity and linear trend. Significance level used was set at 5% (p < 0.05), and the confidence interval was 95%. Data analysis was performed by using SPSS version 21.0. This study was submitted to the research ethics committee of UFCG (CEP/HUAC/UFCG) and was approved under opinion no. 4.487.668.

## RESULTS

The sample consisted of 89 public servants, both permanent and temporary, of both sexes. None of the participants refused to participate in the study (response rate = 100%). Regarding the oral health prevention variables presented in Table 1, slightly more than half of the sample (52.8%) sought and received dental services in the past year, with private services being the most common (46.1%). Reasons for the most recent dental visit included revision/prevention/checkup (34.8%), dental treatment (32.6%) and tooth extraction (28.1%). Most of respondents (51.7%) reported having never been treated at the dental school clinic on their own campus.

**Table 1:** Distribution of the sample of public servants according to sociodemographic variables and oral health prevention, city of Patos, state of Paraíba, Brazil, 2021 (n = 89)

Variables	n (%)
Sex	
Male	51 (57.3)
Female	38 (42.7)
Age (mean) (years)	42
Marital status	
No partner	24 (27.0)
Partnered	65 (73.0)
Color/race	
White	38 (42.7)
Black	8 (9.0)
Brown	43 (48.3)
Schooling (years of study)	
0-9	24 (27.0)
10-12	41 (46.1)
≥ 13	24 (27.0)
Seeking dental care (last 12 months)	
Did not seek dental care	34 (38.2)
I sought and was not attended	4 (4.5)
I sought and was scheduled for another day/place	4 (4.5)
I sought and was attended to	47 (52.8)
Location of last dental appointment	
Public service	26 (29.2)
Private service	41 (46.1)
Health insurance	22 (24.7)
Reason for last dental appointment	
Review/prevention/checkup	31 (34.8)
Pain	4 (4.5)
Tooth extraction	25 (28.1)
Treatment	29 (32.6)
Evaluation of the last dental appointment	
Very good/good	83 (93.3)
Fair	5 (5.6)
Very bad/bad	1 (1.1)
Attendance at the UFCG dental school clinic	
Yes	43 (48.3)
No	46 (51.7)
Self-efficacy score for nightly toothbrushing	
< 20	39 (43.8)
≥ 20	50 (56.2)
Dental-related absenteeism (past 12 months)	
Yes	7 (7.9)
No	82 (92.1)

UFCG = Universidade Federal de Campina Grande.

Respondents were also asked to rate their ability to brush their teeth or dentures at night when they were stressed, depressed, anxious, busy, tired, or worried. This rating was conducted using the nightly toothbrushing self-efficacy scale, with response patterns ranging from completely able to not able at all, and scores ranging from 0 to 24. The results showed that more than half of respondents (56.2%) had a score of 20 or higher (Table 1).

Table 2 shows the distribution of the sample of respondents in terms of subjective and clinical oral health variables. The prevalence of toothache in the last six months was 23.6%, with a mild intensity in 78.7% of cases. Most participants (62.9%) rated their oral health as very good or good. However, a large proportion of respondents (71.9%) reported needing dental treatment at the time of the survey.

**Table 2:** Distribution of the sample of public servants according to subjective and clinical oral health variables, city of Patos, state of Paraíba, Brazil, 2021 (n = 89)

Variables	n (%)
Toothache (past 6 months)	
Yes	12 (23.6)
No	68 (76.4)
Intensity of toothache	
Mild	70 (78.7)
Moderate	7 (7.9)
Severe	12 (13.5)
Pain in the face, on the sides of the head, in the cheeks, or in front of the ears (past 6 months)	
Yes	24 (27.0)
No	65 (73.0)
Intensity of pain in the face, sides of the head, cheeks or in front of the ears	
Mild	67 (75.3)
Moderate	11 (12.4)
Severe	11 (12.4)
Self-assessment of oral health (teeth and gums)	
Very good/good	56 (62.9)
Fair	27 (30.3)
Very bad/bad	6 (6.7)
Self-report of the need for dental treatment today	
Yes	64 (71.9)
No	25 (28.1)
Main reason for needing dental treatment today	
Revision/prevention/check-up	17 (19.1)
Toothache	4 (4.5)
Variables	n (%)
Tooth extraction	8 (9.0)
Root canal treatment	3 (3.4)
Tooth restoration	16 (18.0)
Need for dentures	5 (5.6)
Other	11 (12.4)
Awake bruxism	
Yes	12 (13.5)
No	77 (86.5)
Use of dentures	
Yes	21 (23.6)
No	68 (76.4)
Self-reported need for dentures	
Yes	34 (38.2)
No	55 (61.8)
Number of natural teeth (upper arch)	
< 12	29 (32.6)
≥ 12	60 (67.4)
Number of natural teeth (lower arch)	
< 12	33 (37.1)
≥ 12	56 (62.9)
TMD	
No	55 (58.4)
Mild	28 (31.5)
Moderate	7 (7.9)
Severe	2 (2.2)

TMD (Fonseca anamnestic index) = temporomandibular dysfunction.

Regarding the clinical conditions evaluated, the results showed that the prevalence of awake bruxism was 13.5% and that of TMD was 41.6% (Table 2). In addition, 23.6% of respondents reported the use of dentures. The mean number of natural teeth present in the oral cavity was 12, both in the maxillary and mandibular arches.

In terms of prevalence of the impact of oral health conditions on daily performance, OIDP results showed that at least one performance or daily activity was affected by oral health problems in the 6 months prior to the survey. The most affected activities were “embarrassment or refusal to smile because of teeth” (21.3%), “difficulty eating because of teeth” (20.2%), and “feeling nervous or irritated because of teeth” (18%) (Table 3).

**Table 3:** Prevalence of the impact of oral health conditions on daily performance, according to the OIDP, in the sample of public servants, city of Patos, state of Paraíba, Brazil, 2021 (n = 89)

Items	Yes		No	
	n	%	n	%
1 – Difficulty eating because of teeth	18	20.2	71	79.8
2 – Difficulty speaking because of teeth	3	3.4	86	96.6
3 – Teeth caused discomfort when brushing	11	12.4	78	87.6
4 – Stopped practicing sports because of teeth	1	1.1	88	98.9
5 – Felt nervous or irritated because of teeth	16	18.0	73	82.0
6 – Stopped going out, having fun, attending parties or outings because of teeth	5	5.6	84	94.4
7 – Felt embarrassed or refrained from smiling because of teeth	19	21.3	70	78.7
8 – Interfered with studying/working or doing school/work tasks	5	5.6	84	94.4
9 – Stopped sleeping or slept poorly because of teeth	13	14.6	76	85.4

OIDP = Oral Impacts on Daily Performances.

Regarding the sociodemographic variables, there were significant differences (p < 0.05) between item 9 (“stopped sleeping or slept poorly because of teeth”) and the independent variable gender. The other sociodemographic variables showed no statistical association with OIDP scores (Table 4).

**Table 4:** Association between sociodemographic variables and impact of oral health condition on daily performance, according to the OIDP, in public servants, city of Patos, state of Paraíba, Brazil, 2021 (n = 89)

Variables	Presence of impact (OIDP) (n)								
	Item 1	Item 2	Item 3	Item 4	Item 5	Item 6	Item 7	Item 8	Item 9
Gender						*			*
Male	12	1	9	1	12	5	9	4	11
Female	6	2	2	0	4	0	10	1	2
Marital status									
No partner	2	1	1	0	3	1	3	1	2
Partnered	16	2	10	1	13	4	16	4	11
Color/race									
White	6	1	5	1	7	3	7	3	5
Non-white	12	2	6	0	9	2	12	2	8
Schooling (years)									
0-9	6	1	6	0	5	3	4	1	5
10–12	7	1	4	1	9	1	9	3	6
≥ 13	5	1	1	0	2	1	6	1	2

Pearson’s chi-square test.

OIDP = Oral Impacts on Daily Performances.

*p < 0,05.

Regarding subjective and clinical oral health conditions (Table 5), there were significant differences between the OIDP items and the following variables: item 1 (“Difficulty eating because of teeth”) and the variables toothache (p < 0. 05), intensity of toothache (p < 0.05), self-reported oral health (p < 0.01), awake bruxism (p < 0.01), self-reported need for dentures (p < 0.01); item 2 (“Difficulty speaking because of teeth”) and number of natural teeth in the upper arch (p < 0. 05); item 3 (“Teeth caused discomfort when brushing”) and the variables toothache (p < 0.001), intensity of toothache (p < 0.001), self-reported oral health (p < 0.01), awake bruxism (p < 0.05), number of natural teeth in the lower mouth (p < 0. 05); item 4 (“Stopped practicing sports because of teeth”) and intensity of toothache (p < 0.01); item 5 (“Felt nervous or irritated because of teeth”) and variables toothache (p < 0.001) and intensity of toothache (p < 0.001).

**Table 5:** Association between the impact of oral health condition on daily performance, according to the OIDP, and subjective and clinical variables in public servants, city of Patos, state of Paraíba, Brazil, 2021 (n = 89)

Variables	Presence of impact (OIDP) (n)								
	Item 1	Item 2	Item 3	Item 4	Item 5	Item 6	Item 7	Item 8	Item 9
Toothache (past 6 months)	*		t		t	t		t	t
Yes	8	0	7	1	10	4	7	4	10
No	10	3	4	0	6	1	12	1	3
Intensity of toothache	*		t	t	t	t		t	t
Mild	10	3	4	0	6	1	13	1	3
Moderate	4	0	3	1	4	2	2	2	3
Severe	4	0	4	0	6	2	4	2	7
Self-reported oral health (teeth and gums)	t		t			t	*		t
Very good/good	7	1	3	0	7	2	10	3	4
Fair	7	2	5	1	6	1	5	1	6
Very bad/bad	4	0	3	0	3	2	4	1	3
Awake bruxism	t		*						
Yes	6	1	4	0	3	2	5	1	3
No	12	2	7	1	13	3	14	4	10
Use of dentures									
Yes	3	1	1	0	5	1	5	1	2
No	15	2	10	1	11	4	14	4	11
Self-reported need for dentures	t					*	*		*
Yes	12	2	5	1	7	4	12	3	9
No	6	1	6	0	9	1	7	2	4
Number of natural teeth (upper arch)		*					*		
< 12	9	3	5	1	8	3	10	3	7
≥ 12	9	1	6	0	8	2	9	2	6
Number of natural teeth (lower arch)			*				*		
< 12	6	3	4	1	7	3	11	3	6
≥ 12	12		7	0	9	2	8	2	7
TMD						*		*	
No	10	1	7	1	7	3	7	2	8
Mild	5	1	2	0	6	1	9	1	2
Moderate	2	1	1	0	2	0	2	1	2
Severe	1	0	1	0	1	1	1	1	1

Pearson’s chi-square test.

OIDP = Oral Impacts on Daily Performances. TMD (Fonseca anamnestic index) = temporomandibular dysfunction.

*p < 0.05; tp < 0.001; Φp < 0.01.

In addition, associations were also observed in item 6 (“Stopped going out, having fun, attending parties or outings because of teeth”) and the variables toothache (p < 0.01), intensity of pain (p < 0.01), self-reported oral health (p < 0. 01), self-reported need for dentures (p < 0.05), TMD (p < 0.05); item 7 (“Felt embarrassed or refrained from smiling because of teeth”) and the variables self-reported oral health (p < 0.05), self-reported need for dentures (p < 0. 05), number of natural teeth in the upper arch (p < 0.05), number of natural teeth in the lower arch (p < 0.05); item 8 (“Interfered with studying/working or doing school/work tasks”) and the variables toothache (p < 0.01), intensity of toothache (p < 0. 01), and TMD (p < 0.05); and item 9 (“Stopped sleeping or slept poorly because of teeth”) and the variables toothache (p < 0.001), intensity of toothache (p < 0.001), self-reported oral health (p < 0.01), and self-reported need for dentures (p < 0.05).

## DISCUSSION

In recent years, Dentistry has undergone a transition in which oral health has been recognized as an important determinant of quality of life for various populations. However, few studies have analyzed this perspective in the working class.^2^ The present study clarifies the impact of dental problems on the daily performance of essential functions such as eating, sleeping, speaking, smiling, and even working.

In our study, only gender was associated with the impact of oral disorders among the sociodemographic factors analyzed, a result similar to those reported by Gomes & Abegg.^11^ Self-reported and clinical oral problems were most associated with the presence of impact on the daily activities of employees. For example, toothache and intensity of toothache were variables that exhibited significant statistical differences, confirming other studies that identify toothache as the main issue that negatively affects individuals’ quality of life.^1,7,11,12^

Bruxism and signs of TMD in workers were associated with a lower quality of life because they interfered with daily activities such as eating, going out, and working. These findings are similar to those reported by Pirovani et al.,^13^ who correlated bruxism and TMD with quality of life in adults and concluded they were associated with lower quality of life.

Further, these parameters are important complements to the physical examination for self-reported oral health, both of oral health status and of the need for dental treatment or procedures. They allow the comparison of normative needs with the needs perceived by the individual, which drive the demand for dental services. In fact, seeking dental care may reveal accumulated needs that require more complex treatments that are more likely to have a negative impact on the individual’s daily life. The results of this study show an association between self-reported oral health as fair, very bad, or bad, and self-reported need for dentures, with a negative impact on the activities performed by the respondents.

As far as the clinical conditions are concerned, the average number of teeth present among the workers in this study, both in the upper and in the lower arches, was 12 teeth. This number may explain the presence of toothache as the factor that most contributed to the negative impact on the individuals’ quality of life, since toothache is one of the main reasons for seeking a tooth extraction, leading to a reduction in the number of teeth in the mouth.^14^ On the other hand, the absence of teeth should also be considered as an important factor contributing to the impact on daily activities, as limitations in masticatory function and dissatisfaction with appearance are related to the absence of teeth.^15^

Thus, having less than 12 teeth in the maxilla negatively affected the items “Difficulty speaking because of teeth” and “Felt embarrassed or refrained from smiling because of teeth”. This finding reinforces the relationship between tooth loss and the lack of prosthetic replacement with the negative impact on the individual’s quality of life, especially from an aesthetic perspective. In addition, having fewer than 12 teeth in the lower arch was associated with the negative impact on the “Teeth caused discomfort when brushing” and “Felt embarrassed or refrained from smiling because of teeth” performances, thereby affecting both aesthetics and function.

Limitations of this study include its cross-sectional design, which introduces the possibility of reverse causality, making it impossible to establish causality because it is difficult to determine whether the associations present preceded or occurred after the outcome. In addition, university operations were limited, and remote activities predominated because of the COVID-19 pandemic, resulting in a reduced number of on-campus staff. However, it is extremely relevant to understand the impact of oral health, or the lack thereof, on the daily activities of respondents, a class that is rarely studied, in order to promote better assistance. Therefore, new studies need to be conducted in other work settings to include this population.

## CONCLUSIONS

There was a significant prevalence of impact on the daily activities of public servants because of their oral health status. The variables associated with this impact were: gender, toothache, intensity of toothache, self-reported oral health, awake bruxism, self-reported need for dentures, number of natural teeth in the upper and lower arches, and signs of TMD. Oral problems have a direct impact on physical, psychological, and social performance of the public servants studied.
